# Sequence and expression analysis of the *AMT* gene family in poplar

**DOI:** 10.3389/fpls.2015.00337

**Published:** 2015-05-21

**Authors:** Xiangyu Wu, Han Yang, Chunpu Qu, Zhiru Xu, Wei Li, Bingqing Hao, Chuanping Yang, Guangyu Sun, Guanjun Liu

**Affiliations:** ^1^State Key Laboratory of Tree Genetics and Breeding, Northeast Forestry UniversityHarbin, China; ^2^Department of Plant Nutrition, College of Resources and Environmental Sciences, China Agricultural UniversityBeijing, China; ^3^School of Life Science, Northeast Forestry UniversityHarbin, China

**Keywords:** ammonium transporter, poplar, genome-wide analysis, evolutionary mechanism, expression profile, ammonium deficiency

## Abstract

Ammonium transporters (AMTs) are plasma membrane proteins that exclusively transport ammonium/ammonia. These proteins are encoded by an ancient gene family with many members. The molecular characteristics and evolutionary history of AMTs in woody plants are still poorly understood. We comprehensively evaluated the *AMT* gene family in the latest release of the *Populus trichocarpa* genome (version 3.0; Phytozome 9.0), and identified 16 *AMT* genes. These genes formed four clusters; *AMT1* (7 genes), *AMT2* (2 genes), *AMT3* (2 genes), and *AMT4* (5 genes). Evolutionary analyses suggested that the *Populus AMT* gene family has expanded via whole-genome duplication events. Among the 16 *AMT* genes, 15 genes are located on 11 chromosomes of *Populus*. Expression analyses showed that 14 *AMT* genes were vegetative organs expressed; *AMT1;1/1;3/1;6/3;2* and *AMT1;1/1;2/2;2/3;1* had high transcript accumulation level in the leaves and roots, respectively and strongly changes under the nitrogen-dependent experiments. The results imply the functional roles of *AMT* genes in ammonium absorption in poplar.

## Introduction

For most higher plant species, the main sources of nitrogen are ammonium (NH^+^_4_), nitrate (NO^−^_3_), and amino acids, which are present in the soil as organic and inorganic complexes and compounds (Williams and Miller, 2001). The ammonium transporter (AMT) is responsible for transporting ammonium/ammonia from extracellular into intracellular locations. In plant, once ammonium is uptaked into root cells by AMTs, it is ultimately directed into glutamine via glutamine synthase (GS). Less energy is required for uptake and assimilation of NH^+^_4_ than that of NO^−^_3_ (Bloom et al., 1992). However, a high concentration of NH^+^_4_ can be toxic to plants as indexed by an inhibitory growth (Britto and Kronzucker, 2002).

Recently, some *AMT* genes have been identified and cloned from diverse plant species. Previous studies on phylogenetic analyses of the *AMT* gene family revealed two distinct subfamilies: the *AMT1* subfamily (*AMT1* cluster) and the *AMT2* subfamily (*AMT2/3/4* cluster) (Loqué and von Wirén, 2004; Koegel et al., 2013). The biochemical properties of proteins encoded by *AMT1* cluster genes, and the related regulation mechanisms were reported in the model plant *Arabidopsis thaliana* (Loqué et al., 2007; Yuan et al., 2007, 2009, 2013; Lanquar et al., 2009). The proteins encoded by *AMT1* cluster genes have a high-affinity NH^+^_4_-transport function. For example, both *AtAMT1;1* and *AtAMT1;3* account for 30–35% of the capacity for NH^+^_4_ uptake in nitrogen-deficient roots, and *AtAMT1;2* for 18–26% (Loqué et al., 2006; Yuan et al., 2007). *AtAMT1;4*, which is pollen-specific expressed, contributes to nitrogen nutrition of the pollen via NH^+^_4_ uptake or retrieval (Yuan et al., 2009).

*Populus*, a model system for trees and woody perennial plants, is widely distributed throughout the northern hemisphere. Members of the *Populus* genus are fast-growing trees that are capable of growing under low- or high-NH^+^_4_ and NO^−^_3_ conditions (Min et al., 1999). It is necessary to a better understanding on how the uptake and transport of NH^+^_4_ and NO^−^_3_ are regulated in this genus. In a previous study, 14 *AMT* genes were identified in the *Populus trichocarpa* genome version 1v1. *PtaAMT1;2/1;5/1;6/2;1/2;2* were confirmed to have NH^+^_4_-transporter functions in yeast (Couturier et al., 2007). The expression of *PtaAMT1;2 and PtaAMT3;1* were induced by ectomycorrhiza (Selle et al., 2005; Luo et al., 2009).

In this study, we investigated the evolution and transcription profiles of *Populus AMT* genes by describing the expanded *AMT* gene family consisting of 16 genes, which were dentified in the latest release of the *P. trichocarpa* genome (version 3.0; Phytozome 9.0), analyzing the phylogeny, gene structure, conserved domain, and genome location. Moreover, we comprehensively analyzed the tissue and nitrogen-dependent transcription profiles of *AMT* genes in *Populus*.

## Materials and methods

### Plant seedlings and growth conditions

Cuttings of *P. simonii × P. nigra* were pots-cultivated (organic substrate and vermiculite, 1:1 vol/vol) at Northeast Forestry University Forest Farm, Harbin, China for 3 months under the following conditions; photosynthetic photon flux density (PPFD) of 100 μmol·m-2·s-1, 16-h-light/8-h-dark photoperiod, and 22°C. The plantlets were harvested, and several whole plantlets were frozen in liquid nitrogen and stored at −80°C. New branches were cut into segments of equal length before transferring into modified Long-Ashton medium, pH 5.5 (Dluzniewska et al., 2007). The medium was replaced every 2 days. After 3 weeks, the plantlets were treated with nitrogen at various concentrations. For the nitrogen-free medium, 0.5 mM KNO_3_ and 0.5 mM NH_4_Cl were replaced with 0.5 mM KCl. To supply NH^+^_4_ or NO^−^_3_, the medium contained 2 mM (NH_4_)_2_SO_4_ and 0.5 mM KCl or 4 mM KNO_3_ and 2 mM MgSO_4_, respectively. After the treatments, whole plantlets were harvested, frozen in liquid nitrogen, and stored at −80°C until analysis.

### Identification of *AMT* gene family members in *Populus*

We downloaded the Hidden Markov Model (HMM) profile file (Ammonium_transp.hmm) of the Pfam AMT domain (PF00909) from the Pfam database (Finn et al., 2010). The protein sequences of *P. trichocarpa* were downloaded from Phytozome 9.0 (http://phytozome.jgi.doe.gov/pz/portal.html). We used the HMM modules of PF00909 with HMMER (v 3.0) software to search the proteome of *P. trichocarpa* (Eddy, 2009). Proteins with *e*-values of less than 5E-40 were included in further analyses. Various splicing variants of one gene or incomplete genes were discarded. We searched for the ammonium-domain in all of the collected proteins using Interproscan (http://www.ebi.ac.uk/Tools/pfa/iprscan/) and SMART software (Letunic et al., 2012).

For each putative protein, the grand average of hydropathicity (GRAVY) was calculated using ProtParam (http://web.expasy.org/protparam/). We used TMHMM Server version 2.0 (http://www.cbs.dtu.dk/services/TMHMM/) to predict the transmembrane domains in each AMT protein.

### Phylogenetic analysis and chromosomal location

According to the method of Koegel et al. (2013), we aligned full-length amino acid sequences of AMTs with ClustalW (http://www.ebi.ac.uk/Tools/msa/clustalw2/). The phylogenetic tree was constructed using the Neighbor-Joining (NJ) method and Poisson correction model with MEGA5 software (Tamura et al., 2011). To confirm the reliability of the phylogenetic tree, bootstrap resampling tests were carried out 1000 times.

Information on the chromosomal location of all of the *AMT* genes was downloaded from Phytozome 9.0, and duplicated regions among chromosomes were identified as described by Tuskan et al. (2006). The criterion for tandemly duplicated genes in *Populus* was the occurrence of five or fewer gene loci within a 100-kb region.

### Gene structure and conserved motifs

We used the Gene Structure Display Server (GSDS) program to illustrate the exon/intron organization of individual *AMT* genes (Guo et al., 2007). The Ka/Ks ratio was computed using KaKs_Calculator 2.0 (Wang et al., 2010).

### RNA isolation and quantitative RT–PCR analysis

Total RNA was extracted from leaf, stem, and root tissues using the CTAB method (Chang et al., 1993). The integrity of the extracted RNA was verified by 1.5% agar gel electrophoresis. Approximately 2 μg RNA was used to synthesize first-strand cDNA using the PrimerScript RT Reagent Kit, after removing genomic DNA with gDNAEraser (Takara Biotechnology, Dalian, China). Primer Premier 5.0 (Premier Biosoft, Palo Alto, CA, USA) software was used to design specific primers for semi-quantitative PCR analysis. The primer sequences are listed in Supplementary Table 1. A 7500 Real-Time PCR System (Applied Biosystems) was used to conduct a three-step PCR procedure. In the organ-dependent and nitrogen-dependent expression analyses, transcript levels were normalized to that of the *PtrActin2* gene.

## Results

### Identification of *AMT* genes in *Populus*

By referring to the method of Wang et al. (2004) and Chai et al. (2012), the HMM profile “PF00909” was performed against the *P. trichocarpa* genome to identify *AMT* genes. We ultimately identified 16 putative AMT proteins and the related encoding genes from the *P. trichocarpa* genome. We assigned the names to the 2 *AMT* genes that are not described previously (Table 1). The length of encoded proteins ranged from 458 amino acids (a.a.) to 519 a.a., and their sequences had 7 to 11 trans-membrane domains (TMDs). All of the putative proteins had low GRAVY values (range: 0.369–0.623).

**Table 1:** ***AMT*** **gene family in** ***Populus***.

**S.no**	**Name**	**Accession number**	**Phyotozome**		**Gene**				**Pfam: Ammonium_transp**	
			**Chromosome location**	**ORF(bp)**	**Protein size**	**Gary**	**Exon number**	**TM**	**Location**	***E*-value**
1	PtrAMT1;1	Potri.010G063500	Chr10: 9120743–9122764	1542	513	0.377	1	9	49–473	7.6E-143
2	PtrAMT1;2	Potri.019G023600	Chr19: 2711924–2714239	1521	506	0.365	1	9	45–470	2.2E-140
3	PtrAMT1;3	Potri.008G173800	Chr08: 11862571–11864618	1560	519	0.424	1	9	49–474	1.3E-137
4	PtrAMT1;4	Potri.002G255100	Chr02: 24443271–24444758	1524	507	0.429	1	10	48–473	7.2E-137
5	PtrAMT1;5	Potri.002G255000	Chr02: 24440976–24442512	1506	501	0.486	1	9	50–473	1.8E-134
6	PtrAMT1;6	Potri.009G045200	Chr09: 5126196–5128023	1428	475	0.522	1	9	15–441	1.9E-132
7	PtrAMT1;7^*^	Potri.013G049600	Chr13: 3621326–3622848	1515	504	0.296	2	7	32–455	2.9E-134
8	PtrAMT2;1	Potri.006G102800	Chr06: 7958210–7961388	1494	497	0.485	4	11	24–445	5.5E-84
9	PtrAMT2;2	Potri.016G121400	Chr16: 12596540–12599172	1494	497	0.516	4	11	23–444	1.6E-82
10	PtrAMT3;1	Potri.001G305400	Chr01: 30850782–30853952	1497	498	0.512	3	11	30–454	6.8E-84
11	PtrAMT3;2^*^	Potri.019G000800	Chr19: 130389–137256	1506	501	0.525	3	11	31–455	3.3E-84
12	PtrAMT4;1	Potri.002G047000	Chr02: 3014561–3016477	1398	465	0.527	4	10	24–440	1.8E-82
13	PtrAMT4;2	Potri.018G033500	Chr18: 2675485–2677227	1473	490	0.445	3	11	27–442	1.9E-79
14	PtrAMT4;3	Potri.005G216000	Chr05: 22908162–22910483	1452	483	0.507	3	10	24–440	1.3E-86
15	PtrAMT4;4	Potri.T103600	scaffold_150: 53115–54877	1461	486	0.623	3	10	23–439	1.4E-78
16	PtrAMT4;5	Potri.005G106000	Chr05: 8099969–8101856	1377	458	0.506	3	11	7–419	3.4E-76

* AMT genes of Populus newly identified in this study.

### Phylogenetic and gene structural analyses of *AMT* genes

**Figure 1:**
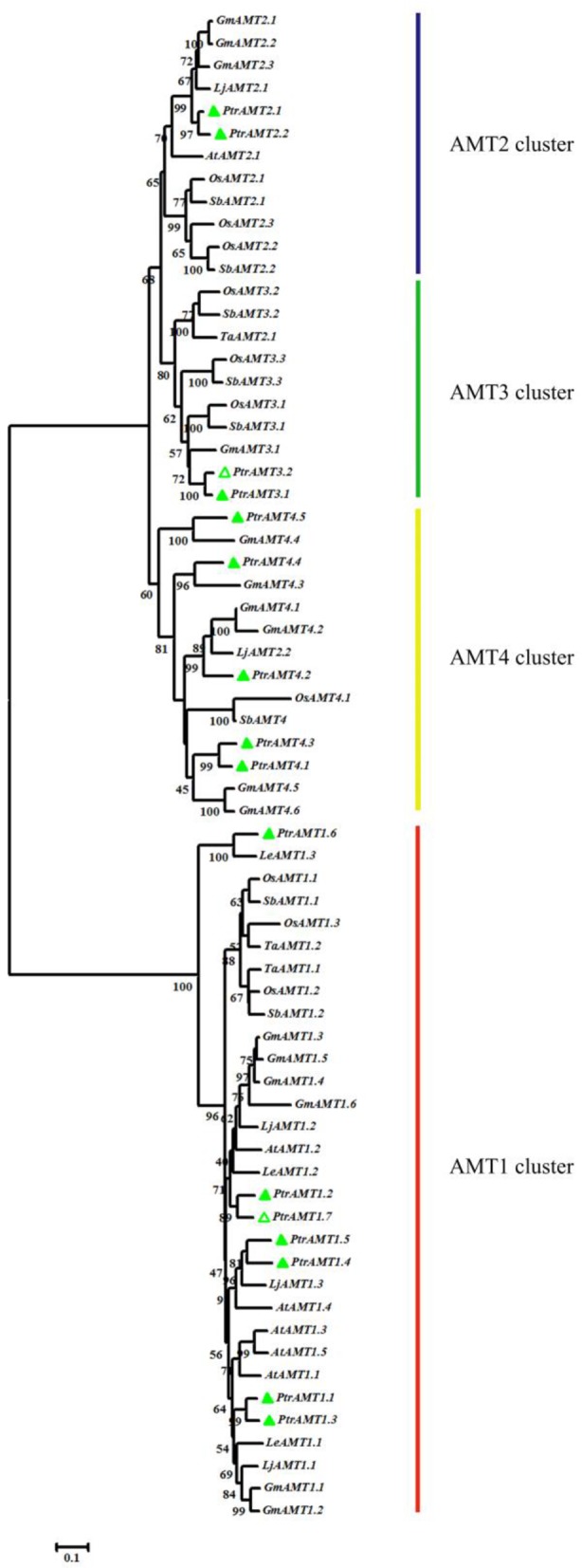
**Phylogenetic tree of proteins encoded by AMT genes from Arabidopsis thaliana, Lycopersicon esculentum, Giycine max, Lotus japonicas, Sorghum bicolor, Oryza sativa, Triticum aestivum, and Populus trichocarpa**. Protein sequences were aligned by Clustalw and tree was constructed by MEGA5 using N-J method, with 1000 bootstrap replicates. Green hollow triangle are the new AMTs in this study.

To evaluate the evolutionary relationships among orthologous *AMT* genes, we constructed a phylogenetic tree with the Neighbor-Joining (N-J) method using MEGA5 software with 8 different plant species (Figure 1). The results revealed two major clades and four clusters. Among the 16 *AMT* genes in *Populus*, 7 genes were in the *AMT1* cluster, and the remaining *AMT* genes were in three other separate clusters (*AMT2*, *AMT3*, and *AMT4*).

**Figure 2:**
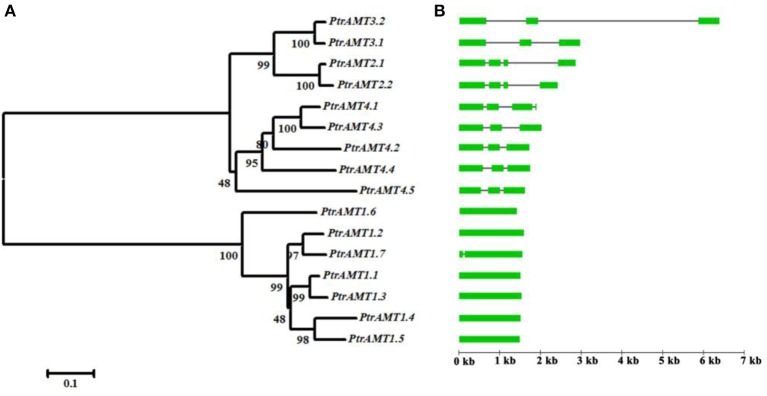
**Paralog phylogenetic tree, gene structures, and motif distribution of** ***Populus AMT*** **gene family**. **(A)** Multiple alignment of full-length AMT protein sequences was conducted using Clustalw. Phylogenetic tree was constructed using MEGA5 by the N-J method, with 1000 bootstrap replicates. **(B)** Gene structures of *AMT* genes. Green boxes show coding exons, black lines show introns.

To investigate the divergence of paralogs and the evolutionary relationships among *Populus* AMT proteins, we aligned full-length sequences of the 16 proteins using ClustalW, and constructed a phylogenetic tree with the Neighbor-Joining method using MEGA5 software (Figure 2A). We identified 6 paralogous pairs, and then determined their substitution rate ratios (non-synonymous vs. synonymous mutations; Ka/Ks). All of 6 paralogous pairs had Ka/Ks ratios of less than 0.5. We deduced that the divergence time of these paralogous pairs ranged from 1.07 to 21.92 million years ago (Table 2). These results indicated that all of the 6 *Populus AMT* gene pairs evolved under the influence of purifying selection.

**Table 2:** **Ka/Ks ratios and estimated divergence time for paralogous** ***AMT*** **genes in** ***Populus***.

**Paralogous pairs**	**Ka**	**Ks**	**Ka/Ks**	**MYA**
1.1 vs. 1.3	0.069	0.326	0.213	17.915
1.4 vs. 1.5	0.109	1.445	0.075	7.945
1.2 vs. 1.7	0.0743	0.399	0.186	21.923
2.1 vs. 2.2	0.033	0.245	0.137	13.465
3.1 vs. 3.2	0.049	0.271	0.183	12.331
4.1 vs. 4.3	0.067	0.246	0.273	13.496

The *AMT* genes in the same cluster had similar exon/intron structures and similar numbers of exons and introns (Figure 2B). Genes in the *AMT1* cluster had 1 exon, except for *PtrAMT1;7* who had 2 exons. And those in the *AMT2* cluster had 4 exons. Genes in the *AMT3* cluster had 3 exons, and those in the *AMT4* cluster had 3 exons, except for *PtrAMT4;1*, which had 4 exons.

**Figure 3:**
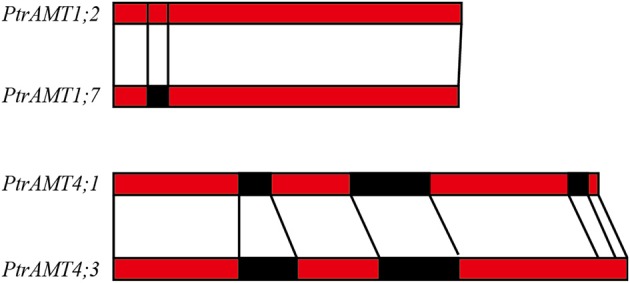
**Duplicated genes in** ***AMT*** **gene family in** ***Populus***. Schematic diagram of gene structure is based on three duplicated paralogous pairs. Exons (red boxes) and introns (black boxes) are shown. Vertical lines show corresponding regions. Numbers of nucleotides are shown beside exons.

We further analyzed the exon/intron structure of the 6 paralogous pairs of *Populus AMT* genes. 4 of the 6 paralogous pairs were well conserved in terms of exon/intron structure, with similar numbers of introns and similar gene lengths. There were greater variations in gene structure among the other 2 paralogous pairs (*PtrAMT4;1/4;3*, and *PtrAMT1;2/1;7*). These differences were rooted in single- and double-intron loss or gain events during the structural evolution of *AMT* paralogs (Figure 3). As shown in Figure 2B, the size of exons was generally well conserved among most members of the 4 *AMT* clusters. Interestingly, Comparing with other members in *AMT2* subfamily, *PtrAMT3;2* had 2 long introns, but CDS sequence was similar to PtrAMT3;1. Therefore, the substantial differences in gene structure resulted from differences in the size of exons and introns among the various genes.

For finding distinctively domain of poplar AMTs, we aligning all the poplar AMT protein sequences with *AtAMT1;1*, *AtAMT2;2* and *EcAmtB* which crystal structures was well characterized (Khademi et al., 2004; Pantoja, 2012). Comparing with *AtAMT1;1*, poplar AMT1 subfamily members also have conserved C-terminal domain and N-terminal domain (Supplementary Figure 1). While *PtrAMT1;6* was similar to *LeAMT1;3* who has a short N-terminal domain. In contrast to all of the TMDs present in *EcAmtB*, all of the poplar AMT gene family members have accordingly conserved TMDs. These results suggested that the *AMT* gene family members are well conserved both in terms of gene structure and specific domain of AMT proteins.

### Chromosomal location and gene duplication of the *Populus AMT* gene family

**Figure 4:**
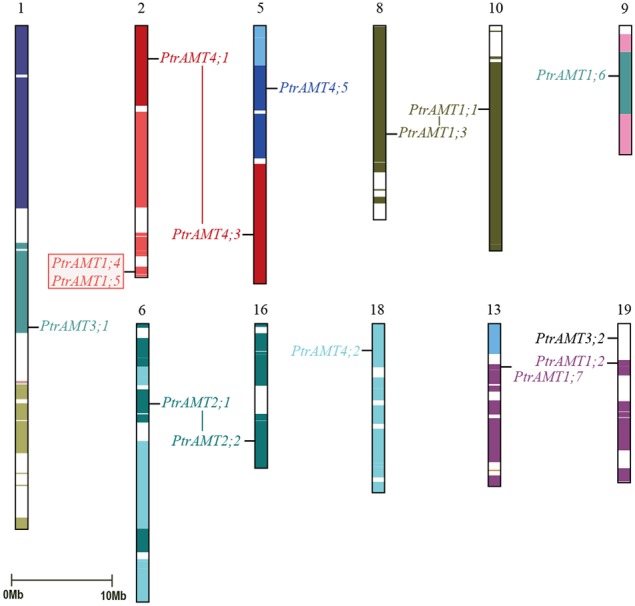
**Chromosomal location and gene duplication of** ***Populus AMT*** **gene family**. Same-colored boxes show segmental duplicated homologous regions. These regions were identified based on duplication coordinates from the *Populus* genome assembly 3.0. Duplicated paralogous pairs of *AMT* genes are connected by colored lines. Red box shows two tandemly duplicated gene pairs.

To explore the relationship between *AMT* genes and segmental duplications in the *Populus* genome, we analyzed the segmental and tandem duplication events in the *AMT* gene family in *Populus*. Based on the location information for *AMTs* in Phytozome 9.0, the genes were marked on the physical map of the *Populus* linkage groups (LG). The *Populus AMT* genes showed a heterogeneous distribution pattern among the chromosomes (Figure 4). We localized 15 of the 16 *AMT* genes on 11 of 19 LG of *Populus*. Only *PtrAMT4;4* was located on unattributed scaffold fragments.

A previous study showed that paralogous segments of the *Populus* genome arose from whole-genome duplication during the salicoid duplication event (Tuskan et al., 2006). In the *AMT* gene family, 14 of the 15 mapped genes were located in duplicated blocks. 4 block pairs harbored 4 paralogous pairs of *AMT* genes (*PtrAMT1;1/1;3*, *PtrAMT2;1/2;2*, *PtrAMT1.2/1.7* and *PtrAMT4;1/4;3*), which arose via a whole-genome duplication event. Paralogous pair *PtrAMT1;4*/*1;5* were arranged in tandem repeats on LG 2 and LG 13, but both lacked corresponding duplicates. Out of 12 *AMT* genes, 2 genes (*PtrAMT3;1*, and *PtrAMT4;5*) also lacked corresponding duplicates. Only *PtrAMT3;2* was not located in duplicated blocks. The corresponding homologs of these genes may have been lost after the duplication event, or genes may have arisen after the salicoid duplication event. In conclusion, duplication events and tandem repeats are expected to contribute to the expansion of the *AMT* gene family in the *Populus* genome.

### Transcription patterns of *Populus AMT* genes in various tissues

**Figure 5:**
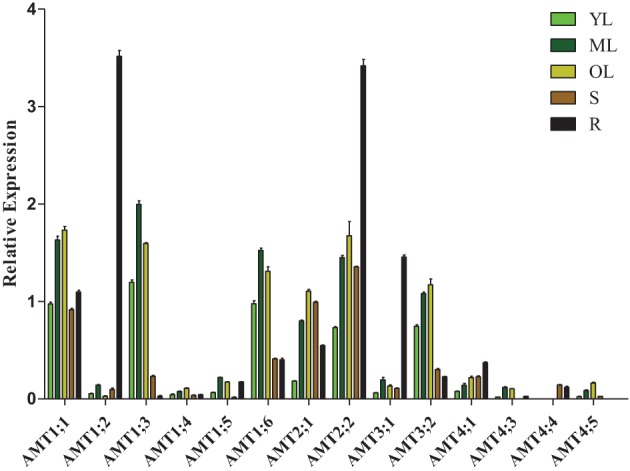
**Relative transcript levels of** ***AMT*** **genes in different tissues of** ***Populus***. YL, young leaf; ML, mature leaf; OL, old leaf; R, root; S, stem. The average expression of each gene was calculated with square root of relative transcript ratio of each gene for display express pattern. Error bars indicate SE.

To investigate the transcription patterns of *Populus AMT* genes during development, we used real-time quantitative RT-PCR to analyze *AMT* gene transcript levels in young leaves, mature leaves, old leaves, stems, and roots of *P. simonii × P. nigra* (Figure 5). Because of significantly difference of transcript accumulation of poplar AMT genes, we used square root value of relative transcript ratio of each gene for display express pattern, and raw date was show in Supplementary Table 4. Finally, we detected transcripts of 14 *AMT* genes: *AMT1;1/1;2/1;3/1;4/1;5/1;6/2;1/2;2/3;1/3;2/4;1/4;3/4;4/4;5*, but there were relatively low transcript levels of *AMT1;4/1;5/3;1/4;1/4;3/4;4/4;5* in the 5 nutritive organs. We detected transcripts of *AMT1;1/1;3/1;4/1;6/2;1/2;2/3;1/3;2/4;1* in all 5 tested tissues. *AMT4;3* leaf-specific transcribed, and *AMT4;4* stem-specific transcribed. There were high transcript levels of *AMT1;3/1;6* in the leaves and *AMT3;1* in the root. However, transcripts of *AMT1;5/4;5* were not detected in the stem or root. A previous study showed that *PtaAMT1;2* was specifically expressed in the root and *PtaAMT1;6/2;1/3;1* in the shoot (Couturier et al., 2007). However under our experiment conditions, all the 4 genes mentioned above were detected in 5 tissues. Among them, *AMT1;6* had high transcript accumulation in leaves, *AMT1;2/3;1* had high transcript accumulation in roots, and *AMT2;1* were expressed similarly in all the 5 tissues, except in young leaves. These differences in transcription patterns may be due to the highly heterozygous genetic background of *P. simonii × P. nigra* and/or differences in experimental conditions.

### *Populus AMT* transcription patterns in response to different nitrogen concentrations

To better understand the function of *AMT* genes in *Populus*, we examined the transcription patterns of poplar *AMT* genes in *P. simonii × P. nigra* under nitrogen-dependent experiment. We selected 10 genes (*AMT1;1/1;2/1;3/1;4/1;5/1;6/2;1/2;2/3;1/3;2*) with high transcript accumulation in the leaf and root to evaluate transcription patterns.

**Figure 6:**
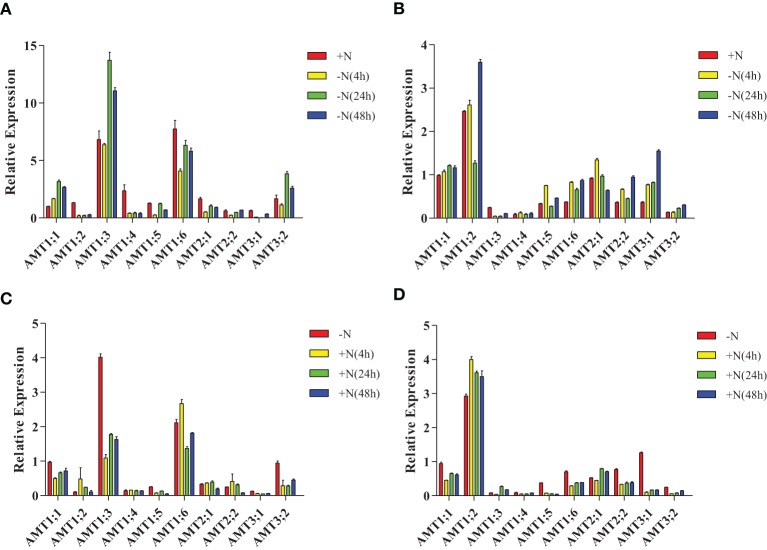
**Expression patterns of** ***Populus AMT*** **genes under nitrogen-starvation and ammonium-resupply conditions**. Plants were grown in modified Long–Ashton medium for 2 weeks, and then transferred to nitrogen-free medium **(A,B)** then, after 2 days, plantlets were transferred to medium with ammonium as the sole nitrogen source for 2 days **(C,D)**. The average expression of each gene was calculated with square root of relative transcript ratio of each gene for display express pattern. Error bars indicate SE.

In leaves of plantlets under nitrogen-starvation conditions, *AMT1;1* was up-regulated, *AMT1;3/3;2* were unchanged and down-regulated, respectively, at 4 h, and then up-regulated at 24 and 48 h. *AMT1;4/1;6/2;1/3;1* were down-regulated, while *AMT1;5* was down-regulated at 4 h, unchanged at 24 h, and further down-regulated at 48 h (Figure 6A, Supplementary Table 5A). In the roots of nitrogen-starved plantlets, *AMT1;1/1;6/2;2/3;1/3;2* were up-regulated; *AMT1;3* was down-regulated; *AMT1;4* was unchanged at this condition. *AMT1;2* was up-regulated at 4 and 48 h but down-regulated at 24 h. *AMT1;2/1;5* was up-regulated at 4 and 48 h but down-regulated at 24 h. *AMT2;1* was up-regulated at 4 h and but down-regulated at 24 and 48 h (Figure 6B, Supplementary Table 5B).

In the leaves of plantlets under NH^+^_4_-resupply conditions, *AMT1;1/1;3/1;5/3;2* were down-regulated and *AMT1;4* was unchanged. *AMT1;6* was up-regulated at 4 h, but down-regulated at 24 h. *AMT2;1/2;2* were up-regulated at 4 and 24 h, but down-regulated at 48 h (Figure 6C, Supplementary Table 5C). In the roots, *AMT1;2* was up-regulated, *AMT1;1/1;5/1;6/ 2;2/3;1/3;2* were down-regulated, and *AMT1;4* was unchanged. *AMT1;3/2;1* was down-regulated at 4 h but up-regulated at 24 h (Figure 6D, Supplementary Table 5D).

**Figure 7:**
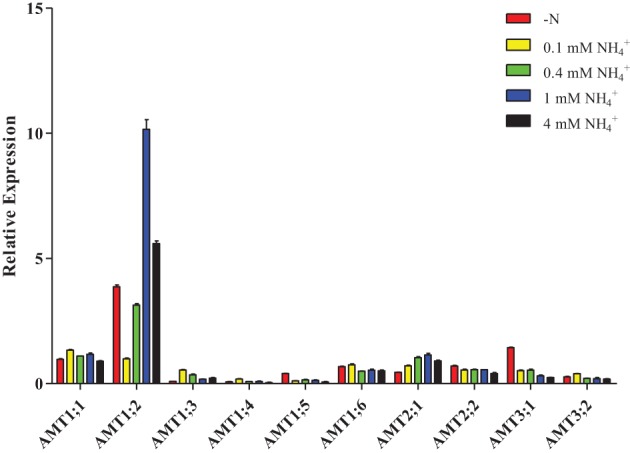
**Relative transcript levels of** ***AMT1;2*** **in roots of** ***Populus*** **in response to different concentrations of ammonium**. Plantlets were grown in nitrogen-free medium for 2 days, and then transferred to medium containing indicated concentrations of ammonium for 1 day. The average expression of each gene was calculated with square root of relative transcript ratio of each gene for display express pattern. Error bars indicate SE.

Interestingly, after plants were resupplied with different concentrations of NH^+^_4_, only *AMT2;1* transcripts had high accumulation when the concentration of NH^+^_4_ was increased in roots (Figure 7, Supplementary Table 6), while the transcripts accumulation of *AMT1;3/1;5/1;6/2;2/3;1* were reduced. The transcription of *AMT1;4/3;2* was up-regulation under 0.1 mM NH^+^_4_ condition, but down-regulated under 0.4, 1, and 4 mM NH^+^_4_, respectively. *AMT1;1* transcripts were up-regulated under 0.1, 0.4, and 1 mM NH^+^_4_conditions, but was down-regulated under 4 mM NH^+^_4_ condition. The expression level of *AMT1;2* was strongly decreased under resupplied 0.1 and 0.4 mM NH^+^_4_ condition, while resupplied 1 and 4 mM NH^+^_4_ led to the transcripts of *AMT1;2* was significantly accumulated.

## Discussion

### *AMT* gene family in *Populus*

We retrieved a total 16 *AMT* genes from the recently released *Populus* genome (Phytozome 9.0, *Populus trichocarpa* 3.0) with improved annotation. Couturier et al. (2007) analyzed an earlier version of the *Populus* genome (1v1) and found 14 *AMT* genes; 6 in the *AMT1* cluster, 2 in the *AMT2* cluster, 1 in the *AMT3* cluster, and 5 in the *AMT4* cluster. In the present study, we found 2 new *AMT* genes in *Populus* (*PtrAMT1;7/3;2*). All of these genes have completely ammonium transport region in their protein sequence.

The evolution of the AMT/MEP/Rh superfamily of integral membrane proteins is extremely complex. Within each of these families, various cases, including duplication and expansion events, gene losses, and horizontal gene transfer events may occur (Couturier et al., 2007; McDonald et al., 2010). In *Populus*, expansion of the *AMT* gene family can be ascribed to duplication events and tandem repeats. In this study, the phylogenetic analysis and chromosomal location information revealed that duplication events, tandem events, and the loss of duplicates after duplication events occurred in the *Populus AMT* gene family. A previous study revealed that the *Populus* genome has undergone two whole-genome duplication events that significantly contributed to the amplification of many multigene families. One of the whole-genome duplication events was the salicoid duplication event that occurred 65 million years ago (Tuskan et al., 2006). Many previous studies have provided evidence for gene duplication in several gene families, including the *GS* gene family and the *NRT* gene family (Castro-Rodríguez et al., 2011; Bai et al., 2013). The ratio of putative *Populus NRT* homologs to corresponding genes in *Arabidopsis* was reported to be 1.4–1.6 (Bai et al., 2013), compared with a ratio of 3.5 for the *AMT* gene family. This result supports the hypothesis that plant species from different environments organize NH^+^_4_ transport with different numbers of NH^+^_4_ transporters (Loqué and von Wirén, 2004).

In the evolutionary history of *Populus*, members of the *AMT* gene family have undergone rigorous selection. The structure of *Populus AMT* genes is well conserved and these genes have different numbers of exons. A previous study reported that most genes in the *AMT1* cluster have one exon and no introns, except for *LjAMT1;1*, which has an intron in its open reading frame (ORF) (Salvemini et al., 2001). In *Populus*, *PtrAMT1;7* also has an intron in ORF, but could not detected it in all the nutritive organ, it may express in specific tissue.

In AMT gene family, function of extracellular N-terminus play a role for oligomer stability. In *Lycopersicon esculentum*, *LeAMT1;1/1;2* were detected as a trimeric complex in *planta*, but in the paraloge *LeAMT1;3* who had a short N-terminus, trimeric complexes were not detected (Graff et al., 2011). This may indicate that *PtrAMT1;6* is similar to *LeAMT1;3* who maintain dimer and monomer complexes on plasma membrane. Previous studies on *AtAMT1;1* showed that protein activity could be controlled by phosphorylation site T460, which was localized in C-terminus conserved domain (Loqué et al., 2007; Lanquar et al., 2009). When compared with *AtAMT1;1*, all the members of poplar AMT1 subfamily members have the conserved phosphorylation site T, except *PtrAMT1;6* whose site was replaced by S; but this site was not conserved in AMT2 subfamily members (Supplementary Figure 1). These results may indicate phosphorylation at specific site of poplar AMT1 may be equally important for regulate ammonium up-take under various external environment conditions, and there were possible different regulation mechanisms between AMT1 and AMT2 subfamily members.

### Transcript profiles of *AMT* genes in *Populus*

In the *Arabidopsis* roots, the genes in the *AMT1* cluster encode proteins responsible for NH^+^_4_ uptake (Yuan et al., 2007). *AtAMT1;1/1;2/1;3/2;1* account for 90% of high-affinity NH^+^_4_-uptake capacity in the root, while *AtAMT1;4* is responsible for the high-affinity NH^+^_4_-uptake capacity of pollen (Loqué et al., 2006; Yuan et al., 2007, 2009). However, in poplar, the physiological function of AMTs is still not well known. When comparing with *Arabidopsis*, there are more *AMT* gene family members in poplar than in *Arabidopsis*. These may indicate function redundancy of AMTs in poplar; or execute special function depend on differential tissue expression, like AtAMT1;3 who can mediate lateral root branching (Lima et al., 2010).

In this study, transcripts of 14 *AMT* genes were detected in nutritive organs. There were relatively high transcript levels of *AMT1;1/1;3/1;6/2;1/2;2/3;2* in the leaves, *AMT1;1/2;1/2;2* in the stems, and *AMT1;1/1;2/2;2/3;1* in the roots. These results indicate that these genes may play different physiological functions in ammonium utilization. Based on our observations, we propose that *AMT1;1/1;2/2;2/3;1* may be suspected to be responsible for ammonium uptake from the soil; and the others may be involved in ammonium redistribution, for example, *AMT1;1/2;1/2;2* may play key roles in the ammonium transport from roots to shoots, *AMT1;6* may participate in the retrieval and import from apoplast of leaves (von Wirén et al., 2000), and *AMT1;1/1;3/1;6/2;1/2;2/3;2* may be in charge of ammonium retrieval from old leaves to young leaves (Couturier et al., 2007). Noteworthy, paralogous pairs *PtrAMT3;1*/*3;2* had different intron length and express pattern and in roots and leaves, these results indicate that these two genes may have different transcriptional regulation mechanism and/or different function in specially tissue or cell.

A comprehensive analysis of RNA-seq data and Microarray data (Yang et al., 2008) from popgenie v3 (http://www.popgenie.org/) confirms that *AMT1;2* prefers to be expressed in roots, but less in the leaves and stems, while *AMT1;6* prefer to be expressed in leaves, but had low expression level in the stems and roots (Supplementary Tables 2, 3).

In this study, we propose that the ammonium-dependent expression of some *PtrAMTs* may be controlled by a local ammonium signal in roots or a systematic N signal in leaves, respectively. The expression pattern of *AMT1;1/2;2/3;1* have acutely change under nitrogen starvation and ammonium supply in roots, these results are similar to *ZmAMT1;1a/1;3* who could be controlled by a local ammonium signal (Gu et al., 2013), and this expression pattern may improve NH^+^_4_ up-take efficiency. Although *AMT1;2* showed up-regulated transcription under nitrogen starvation and NH^+^_4_-resupply conditions, but the external ammonium concentration can affect *AMT1;2* transcript levels in roots, this result suggests that *AMT1;2* may play a housekeeping role in root who is always ready to transport ammonium in the roots. In addition, *AMT1;1/1;2/2;2/3;1* had a high expression level in roots, they may make up the loss of ammonium transport in the roots of some down-regulated AMT genes under nitrogen-dependent experiment. Under nitrogen starvation and ammonium supply condition, transcripts accumulation of *AMT1;1/1;3/3;2* may regulate by the whole-plant N status, who had high transcripts accumulation after nitrogen starvation for 24 h. While *AMT1;6* had oppositely expression pattern under nitrogen starvation, these results may indicate that expression of *AMT1;6* was controlled by ammonium concentration in the apoplast of leaves.

### Conflict of interest statement

The authors declare that the research was conducted in the absence of any commercial or financial relationships that could be construed as a potential conflict of interest.
