# Supplementary material for: Sequence and expression analysis of the AMT gene family in poplar
Source: Front Plant Sci. 2015 May 21;6:337. doi: 10.3389/fpls.2015.00337 (PMC4440354; doi:10.3389/fpls.2015.00337)
Supplement: Supplementary file 1 [file Presentation1.PDF]

Supplementary Table 1: Primers used for quantitative RT–PCR analysis.

Gene Name	Primer-Forward(5'-3')	Primer-Reverse(5'-3')
Actin-2	CACAACTGCTGAACGGGAAAT	CAGGGCAACGGAAACACTCT
AMT1;1	GGTTTACCCGAACAAACCGAATC	CTCATCCTCAGATGAGATCCTC
AMT1;2	CATAACCTTTGGGCACATCTGTCT	GCTGGATATGCTGGTGAACTGG
AMT1;3	GGGAATCTCCTGGCTGCTCATT	CTCTGGGACTCGTCGTCATCGT
AMT1;4	CCTATTGTGGTTCGGGTGGTAT	AGTGTTGTTGTCACGGCTGTTC
AMT1;5	AGGTTGTTAGCTGGTCACTGGA	GGATCATCATAATGGAACTTCTCAG
AMT1;6	TGGGCTCAACGTCTTGGCTCT	TCTCACCACTCCCGCCTCACCT
AMT1;7	GGAGCGGTCGGTCCGTGGTATT	AAGGTAGTAAGGGCTGCTGTGC
AMT2;1	CTTGGTATCCGATGGCCTCACT	ATGATAAAGAAATCCGCCTCCC
AMT2;2	GAACATAGTGTCAACAACGCTAATCC	CCTCTCCAAACAAGGTGGTAATCC
AMT3;1	AGGGTGATAACTCATGGCAAATG	GTACAACAGCAGCGAAAGCATAT
AMT3;2	AAGCCCTTGCGTCCATTTCCTT	TGCCACTGGGACTGGGTTAGGT
AMT4;1	GCTGCTTTGGATGGGATGGAGT	CCACGGGATGCTACCAGACATT
AMT4;2	CTGGGGCAATGGAGACAGAC	TTCTCAGCTTGACGAGCAGCC
AMT4;3	CCGCTGCAGGACTCAAGCAA	ACTTGAGGGAACTCTTCAACACC
AMT4;4	CCGCAGCATATTGGGTGGGT	AAGCAAGCCTGCTCCTGCAAGC
AMT4;5	TGGTGCTGTCCAGGGGATGA	GTGCTGCCCATGGTTCCACT

Supplementary Table 2: Microarray relative expression Log2 ratio of AMT1;2 and AMT1;6 from popgenie.

	Young-leaves	Mature-leaves	Nodes	Internodes	Roots
AMT1;2	0	-0.03	-0.05	0.01	3.51
AMT1;6	1.81	2.11	0	-0.26	-1.06

Supplementary Table 3: RNA-seq relative expression Log2 ratio of AMT1;2 and AMT1;6 from *popgenie*.

	Leaves Young Expanding	Leaves Mature	Wood	Roots
AMT1;2	1.22	-0.4	2.41	4.56
AMT1;6	-0.29	0.94	-0.01	-1.09

Supplementary Table 4: Relative transcript levels of AMT genes in different tissues of Populus. YL young leaf; ML: mature leaf; OL: old leaf ; R root; S:stem.

	YL		ML		OL		S		R	
	Relative expression	SE	Relative expression	SE	Relative expression	SE	Relative expression	SE	Relative expression	SE
AMT1;1	0.32	0.012711	0.882391	0.041452	0.995908	0.042698	0.278137	0.007804	0.397636	0.014338
AMT1;2	N/A	N/A	N/A	N/A	N/A	N/A	0.003117	0.000963	4.104259	0.137825
AMT1;3	0.475948	0.017743	1.321358	0.048456	0.844781	0.00827	0.017668	0.001768	0.000286	0.000134
AMT1;4	0.000667	0.00023	0.001915	0.000295	0.003915	0.000445	0.000443	0.000151	0.000636	8.66E-05
AMT1;5	0.001444	0.000116	0.016079	0.000462	0.009893	0.000506	N/A	N/A	0.010195	0.000271
AMT1;6	0.316239	0.020024	0.770587	0.025543	0.570697	0.038432	0.056082	0.001866	0.053615	0.004066
AMT2;1	0.011109	0.000494	0.212711	0.003768	0.405262	0.012233	0.325912	0.00669	0.099666	0.002134
AMT2;2	0.1777	0.004316	0.698326	0.021681	0.935631	0.159048	0.609171	0.004997	3.8776	0.149213
AMT3;1	0.001291	0.000155	0.012668	0.003194	0.005826	0.00104	0.003879	0.0003	0.70433	0.020717
AMT3;2	0.184424	0.007462	0.386443	0.011696	0.457034	0.043895	0.030308	0.002043	0.017181	0.00049
AMT4;1	0.00197	0.000249	0.006653	0.001834	0.015856	0.002276	0.017107	0.001431	0.046122	0.001817
AMT4;3	0.000109	6.35E-06	0.004639	0.00065	0.003579	0.000235	N/A	N/A	0.000243	3.23E-05
AMT4;4	N/A	N/A	N/A	N/A	N/A	N/A	0.006826	0.000312	0.004687	0.001008
AMT4;5	N/A	N/A	0.002428	0.000414	0.008932	0.000662	N/A	N/A	N/A	N/A

Supplementary Table 5: Expression patterns of Populus AMT genes under nitrogen-starvation and ammonium-resupply conditions. Plants were grown in modified Long–Ashton medium for 2 weeks, and then transferred to nitrogen-free medium; then, after 2 days, plantlets were transferred to medium with ammonium as the sole nitrogen source for 2 days.

Supplementary Table 5A: Leaf samples under nitrogen-free medium condition.

	-N		+N(4h)		+N(24h)		+N(48h)	
	Relative expression	SE	Relative expression	SE	Relative expression	SE	Relative expression	SE
AMT1;1	0.065357	0.004173	0.185204	0.005391	0.655483	0.060119	0.465939	0.021478
AMT1;2	0.113436	0.00475	0.002424	0.001166	0.002734	0.000998	0.005069	0.001503
AMT1;3	3.079431	0.695619	2.648015	0.098685	12.33617	1.27742	7.986021	0.39984
AMT1;4	0.384785	0.153727	0.010602	0.002327	0.012532	0.003784	0.009939	0.003976
AMT1;5	0.107649	0.003351	0.004475	0.000712	0.101215	0.007843	0.031542	0.002235
AMT1;6	3.958993	0.763314	1.09978	0.092141	2.619923	0.374571	2.2185	0.202445
AMT2;1	0.179437	0.031684	0.017583	0.002053	0.072614	0.012995	0.05409	0.005975
AMT2;2	0.024737	0.007526	0.003675	0.000684	0.014798	0.001439	0.030005	0.001279
AMT3;1	0.026366	0.004166	0.000422	1.75E-05	3.73E-05	2.68E-05	0.007466	0.001313
AMT3;2	0.187636	0.070598	0.084084	0.013773	0.970655	0.101949	0.443301	0.054637

Supplementary Table 5B: Root samples under nitrogen-free medium condition.

	+N		-N(4h)		-N(24h)		-N(48h)	
	Relative expression	SE	Relative expression	SE	Relative expression	SE	Relative expression	SE
AMT1;1	0.331502	0.011554	0.394	0.026341	0.499554	0.016101	0.460151	0.039439
AMT1;2	2.071976	0.028928	2.327849	0.188708	0.549809	0.044592	4.410007	0.174727
AMT1;3	0.019711	0.001968	0.000592	0.000183	0.000633	0.000294	0.003778	0.000452
AMT1;4	0.002713	0.001265	0.005261	0.001864	0.002686	0.000968	0.004052	0.001543
AMT1;5	0.038206	0.001899	0.192489	0.004449	0.025459	0.00154	0.072496	0.002756

AMT1;6	0.047197	0.002022	0.232096	0.010047	0.147042	0.016621	0.254851	0.018197
AMT2;1	0.2876	0.010266	0.617578	0.031998	0.319292	0.020236	0.139612	0.005623
AMT2;2	0.04542	0.00216	0.150132	0.006035	0.068401	0.004234	0.304937	0.021828
AMT3;1	0.045447	0.003861	0.203077	0.007444	0.23104	0.004284	0.816376	0.03481
AMT3;2	0.006231	0.000685	0.006077	0.001488	0.017794	0.001065	0.031534	0.001381

Supplementary Table 5C: Leaf samples under ammonium resupply condition.

	-N		+N(4h)		+N(24h)		+N(48h)	
	Relative expression	SE	Relative expression	SE	Relative expression	SE	Relative expression	SE
AMT1;1	0.9408993	0.0433717	0.245076	0.0251859	0.438618	0.0326682	0.5141606	0.1168649
AMT1;2	0.0102365	0.0030355	0.3392137	0.2754843	0.0576084	0.0022565	0.0128008	0.0085662
AMT1;3	16.126673	0.8074217	1.1987305	0.2272548	3.1428134	0.1013458	2.6607515	0.2631932
AMT1;4	0.02007	0.0080283	0.0256989	2.254E-05	0.0199162	0.004156	0.0183403	0.0024016
AMT1;5	0.0636951	0.0045124	0.006188	0.0008295	0.0157926	0.0027657	0.0022714	0.0010125
AMT1;6	4.4799561	0.4088091	7.1316159	0.6372362	1.8707455	0.1566056	3.2934153	0.0574545
AMT2;1	0.1092265	0.0120653	0.1328205	0.0066892	0.1537615	0.0303611	0.0376004	0.0095797
AMT2;2	0.0605904	0.0025838	0.2160644	0.2129284	0.0960942	0.0189353	0.0069014	0.0007813
AMT3;1	0.0150767	0.0026506	0.0033805	0.0009827	0.0026868	7.235E-05	0.0034625	0.0009223
AMT3;2	0.8951862	0.1103326	0.1080041	0.1105151	0.0784746	0.0134387	0.2069756	0.025612

Supplementary Table 5D: Roots samples under ammonium resupply condition.

	-N		+N(4h)		+N(24h)		+N(48h)	
	Relative expression	SE	Relative expression	SE	Relative expression	SE	Relative expression	SE
AMT1;1	0.895322	0.076738	0.204192	0.006325	0.425334	0.019671	0.371847	0.039978
AMT1;2	8.580615	0.339969	16.0265	0.713631	13.03831	0.372812	12.27246	1.167188

AMT1;3	0.007351	0.00088	0.001736	0.000249	0.071546	0.008992	0.029396	0.002997
AMT1;4	0.007884	0.003002	0.002504	0.000972	0.002857	0.001075	0.006411	0.000918
AMT1;5	0.141056	0.005363	0.005918	0.000963	0.003377	0.001099	0.002309	0.00019
AMT1;6	0.495867	0.035406	0.085083	0.002681	0.143698	0.009201	0.151084	0.000907
AMT2;1	0.271645	0.010941	0.200064	0.003629	0.630208	0.016114	0.496662	0.027637
AMT2;2	0.593321	0.04247	0.111838	0.003022	0.137233	0.023337	0.149888	0.018004
AMT3;1	1.588434	0.06773	0.011723	0.001622	0.02778	0.002776	0.028835	0.003181
AMT3;2	0.061355	0.002687	0.003589	0.000562	0.006693	0.001282	0.022209	0.001129

Supplementary Table 6: Relative transcript levels of AMT1;2 in roots of Populus in response to different concentrations of ammonium. Plantlets were grown in nitrogen-free medium for 2 days, and then transferred to medium containing indicated concentrations of ammonium for 1 day.

	-N		0.1 mM NH4+		0.4 mM NH4+		1 mM NH4+		4 mM NH4+	
	Relative expression	SE	Relative expression	SE	Relative expression	SE	Relative expression	SE	Relative expression	SE
AMT1;1	0.925445	0.058938	1.79817	0.046876	1.203148	0.015115	1.367265	0.115378	0.790789	0.04439
AMT1;2	14.93921	0.57743	0.968861	0.076233	9.806501	0.371789	103.275	7.958496	31.34217	1.117771
AMT1;3	0.007832	0.001056	0.29013	0.028513	0.120666	0.022278	0.029975	0.002899	0.048124	0.00967
AMT1;4	0.004747	0.002283	0.031331	0.006324	0.005641	0.00135	0.007793	0.001774	0.001641	0.001973
AMT1;5	0.15985	0.006463	0.011773	0.00155	0.020706	0.005448	0.016541	0.003865	0.004687	0.002705
AMT1;6	0.458936	0.017791	0.563131	0.059944	0.244598	0.006125	0.287844	0.046634	0.263722	0.030112
AMT2;1	0.204566	0.006295	0.500727	0.036904	1.065073	0.092162	1.310905	0.151762	0.82529	0.046105
AMT2;2	0.486327	0.050161	0.295395	0.038418	0.306297	0.024055	0.302764	0.007131	0.161445	0.028686
AMT3;1	2.04526	0.061563	0.270964	0.020706	0.28267	0.051505	0.093696	0.022467	0.055173	0.004211
AMT3;2	0.072033	0.01346	0.154677	0.005712	0.044582	0.003241	0.039715	0.018612	0.032908	0.005253

Supplementary Figure 1: Alignment of 19 AMT transporters. Red boxes are TMDs, blue box is the C-terminals conversed domain of AtAMT1;1 and yellow box is the N-terminals of LeAMT1;3.

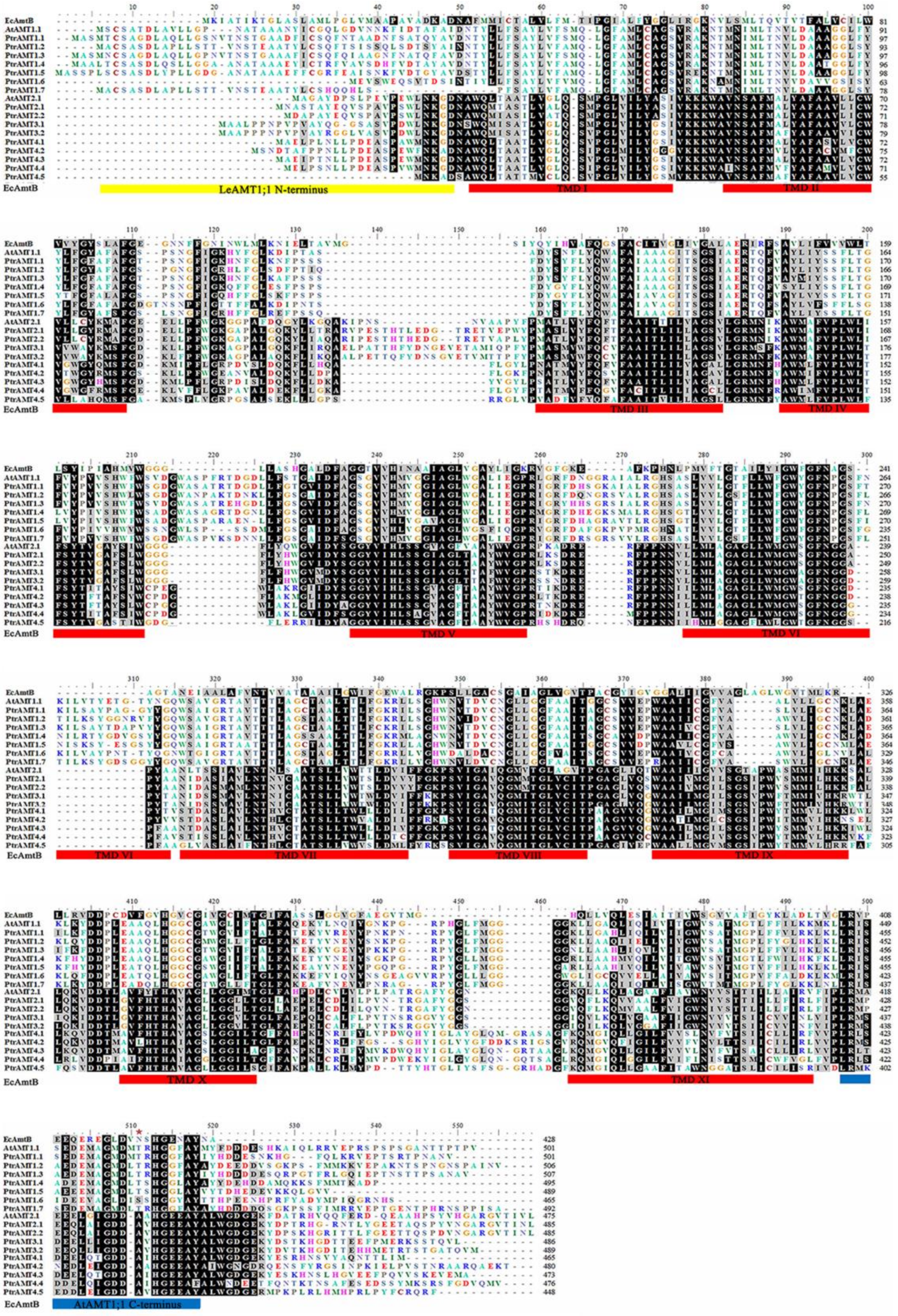

Supplementary Figure 1

Methods S1

The accession numbers or gene models quoted from Couturier et al., (2007) and Koegel et al., (2013).

Escherichia coli from Uniprot : EcAMTB (P69681);

Arabidopsis thaliana (TAIR 10): AtAMT1;1 (At4g13510), AtAMT1;2 (At1g64780), AtAMT1;3 (At3g24300), AtAMT1;4 (At4g28700), AtAMT1;5 (At3g24290), AtAMT2;1 (At2g38290);

Lycopersicon esculentum from Uniprot: LeAMT1;1 (P58905), LeAMT1;2 (O04161), LeAMT1;3 (Q9FVN0);

Lotus japonicas from Uniprot: LjAMT1;1 (Q9FSH3), LjAMT1;2 (Q7Y1B9), LjAMT1;3 (Q70KK9), LjAMT2;1 (Q93X02);

Glycine max Phytozome 6.0 database: GmAMT1.1 (Glyma20g21030.1), GmAMT1;2 (Glyma10g26690.1), GmAMT1;3 (Glyma10g31080.1), GmAMT1;4 (Glyma10g31110.1), GmAMT1;5 (Glyma1031130.1), GmAMT1;6 (Glyma20g36390.1), GmAMT2;1 (Glyma07g18670.1), GmAMT2;2 (Glyma18g43540.1), GmAMT2;3 (Glyma01g30920.1), GmAMT3;1 (Glyma05g33010.1), GmAMT4;1 (Glyma09g41810.1), GmAMT4;2 (Glyma20g00680.1), GmAMT4;3 (Glyma19g43380.1), GmAMT4;4 (Glyma02g04960.1), GmAMT4;5 (Glyma02g16200), GmAMT4;6 (Glyma10g03600.1).

Oryza sativa from Uniprot: OsAMT1;1 (Q7XQ12), OsAMT1;2 (Q6K9G1), OsAMT1;3 (Q6K9G3), OsAMT2;1 (Q84KJ7), OsAMT2;2 (Q8S230), OsAMT2;3 (Q8S233), OsAMT3;1 (Q84KJ6), OsAMT3;2 (Q851M9), OsAMT3;3 (Q69T29), OsAMT4;1 (Q10CV4);

S. bicolor genome (v1.1) database: SbAMT1;1 (Sb06g022230), SbAMT1;2 (Sb04g026290), SbAMT2;1 (Sb09g023030), SbAMT2;2 (Sb03g038840), SbAMT3;1 (Sb03g041140), SbAMT3;2 (Sb01g001970), SbAMT3;3 (Sb04g022390), SbAMT4 (Sb01g008060);

Triticum aestivum (Uniprot): TaAMT1;1(Q6QU81), TaAMT1;2(Q6QU80),

TaAMT1;3(Q6T8L6).

Couturier, J., Montanini, B., Martin, F., Brun, A., Blaudez, D., & Chalot, M. (2007). The expanded family of ammonium transporters in the perennial poplar plant. New Phytologist, 174(1), 137-150.

Koegel, S., Ait Lahmidi, N., Arnould, C., Chatagnier, O., Walder, F., Ineichen, K., & Courty, P. E. (2013). The family of ammonium transporters (AMT) in Sorghum bicolor: two AMT members are induced locally, but not systemically in roots colonized by arbuscular mycorrhizal fungi. New Phytologist, 198(3), 853-865.
